# Twelve Years of Experience in the Management of Testicular Germ Cell Tumors at a Referral Center in Portugal

**DOI:** 10.4021/wjon245w

**Published:** 2010-11-02

**Authors:** Diana Valadares, Filipe Nery, Franklim Marques

**Affiliations:** a Department of Internal Medicine, Centro Hospitalar do Porto - Hospital Santo Antonio, Porto, Portugal; b Director of Oncology Services, Centro Hospitalar do Porto - Hospital Santo Antonio, Porto, Portugal

**Keywords:** Testicular germ-cell tumors, Risk factors, Incidence, Outcome

## Abstract

**Background:**

Testicular germ cell tumors (TGCT) are generally rare but quite frequent in young males. Guidelines are well established for their management.

**Methods:**

We present the first report from Portugal on clinical, histological, treatment modalities and outcomes of a population with TGCT. Data was retrospectively analyzed for the 1996 through 2008 period, applying a previous internally validated protocol.

**Results:**

Seventy nine patients with TGCT were identified, 40.5% had seminomatous and 59.5% nonseminomatous tumors. Incidence rates were higher among males in their twenties and thirties. Pain and swelling testis were the most common symptoms and microlithiasis was detected in 20.3% of patients. Lower stages were more frequent in seminomatous tumors. Orchiectomy was done in all patients and further therapy was performed by guidelines recommendations in 86.1% of them. Hematological toxicity was found in 44.3% of the population studied and free disease survival rates were at 88.6%.

**Conclusions:**

This retrospective study corroborates the European Western country trends concerning TGCT. Mortality was only seen in nonseminomatous TGCT group. Good risk and lower TGCT stages have no deaths reported. Public health campaigns should be undertaken to guide patients to seek medical advice earlier in the course of the disease.

## Introduction

Testicular germ cell tumors (TGCT) are a rare malignant disease, counting for 1 to 2% of all male cancers. However, they are the most common malignancy in the younger generations [1]. In Europe, it seems that incidence has been rising in the past years with decreasing mortality rates. In Portugal, the incidence of TGCT is 2.3/100,000, below the European average of 4.2/100,000, with mortality rates of 0.3/100,000, which seem to be increasing [2, 3]. TGCT has a very good prognosis and even in metastatic disease the cure rates are near 80% [4].

There are many known risk factors to develop TGCT (previous contralateral TGCT, family history, cryptorchidism, klinefelter syndrome, etc) [5]. International guidelines are available to facilitate correct diagnosis, treatment and follow-up [6].

The authors intend to present the experience in managing TGCT over 12 years, at the Department of Oncology in Centro Hospitalar do Porto, Hospital Santo Antonio in Portugal. They analyzed risk factors for TGCT, stage at the time of diagnosis, histological characteristics, management, treatment options and survival. To our knowledge this is the first report ever done in Portugal, to assess the country’s status and ranking in international setting.

## Materials and Methods

From pathology department cancer database of our hospital, 85 testicular cancers were recorded between 1996 and 2008. A total of 79 cases were identified as being testicular germ cell tumors. From the six cases excluded, two were non-Hodgkin lymphomas, one Leyding-cell tumor, one leiomyosarcoma, one liposarcoma and one sex cord-stromal tumor.

All the patients identified were treated and followed in our center. All the files were analyzed, applying a protocol covering risk factors, presentation form, initial stage, surgical procedure, histology, concomitant treatment and response, time free of disease, relapse, salvage treatment whenever done, lateral effects, mortality (cancer and non-related mortality). Staging was done using the TNM system created by the American Joint Committee on Cancer (AJCC). Tissue specimens were reviewed in our center and World Health Organization (WHO) classification for testicular cancer was applied. Patients were broadly divided in two subgroups, i.e., seminomas (STGCT) and nonseminomas (NSTGCT).

All the patients remained under surveillance in our center and latest medical visit of each one was recorded in current year (2010) to extrapolate survival.

Statistical data were computed and analyzed using SPSS version 17.0 software. Descriptive statistics were used to characterize the most relevant clinical parameters. Actuarial survival rates were estimated using the Kaplan-Meier product limit method, and differences observed among patient subgroups were assessed by the log-rank test.

## Results

**Figure 1:**
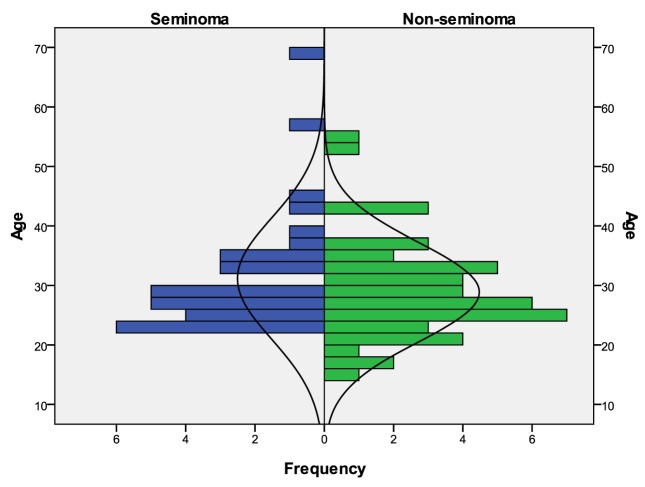
Age distribution considering both subgroups: STCGT and NSTCGT.

A total of 79 male patients were identified as having TGCT. From those, 32 (40.5%) were STCGT and 47 (59.5%) NSTGCT. Overall mean age at diagnosis was 29.8 years old, with the youngest boy being 15 years old, and the oldest patient being 69 years old. Mean age at diagnosis for seminomas was 31.2 and 28.9 years old for nonseminomas, peaking in their twenties and thirties (Fig 1). Two patients were HIV and six HCV positive.

Microlithiasis occurred in 20.3% of the patients. Cryptorchidism, infertility and previously testicular surgery each counted for 3.8%. Previous testicular cancer, testicular atrophy and varicocele were present in 2.5%.

The most common symptoms at presentation were testicular pain and swelling testis in identical percentages in both groups. Symptomatic metastasis was more frequently found in nonseminomatous TGCT (Table 1).

**Table 1:** Clinical Symptoms at Diagnosis in STGCT and NSTGCT

	Symptoms	STGCT (%)	NSTGCT (%)
Localized	Testicular atrophy	0	1 (2.1%)
	Swelling testis	6 (18.7%)	9 (19.1%)
	Pain	9 (28.1%)	14 (29.8%)
	Post traumatic pain	0	1 (2.1%)
	Breast enlargement	0	4 (8.5%)
Disseminated	Right atrium thrombus	0	1 (2.1%)
	Gastro-intestinal	0	2 (4.3%)
	Retroperitoneal ganglionar	1 (3.1%)	8 (17.0%)
	Lumbar pain	2 (6.2%)	5 (10.6%)
	Bone pain	1 (3.1%)	2 (4.3%)
	Central nervous system	1 (3.1%)	0
	Leg swelling	0	1 (2.1%)

Referral to the CHP was mostly done by general practitioners (59.5%), followed by patients who sought medical attention at the emergency department due to some of the symptoms mentioned above (35.4%) and finally 5.1% of the patients were sent by physicians of neighboring hospitals.

All of the patients underwent testicular ultrasound, thoraco-abdomino-pelvian CT scan and tumoral markers (α-fetoprotein (AFP), β-human chorionic gonadotrophin (β-hCG) and lactic dehydrogenase (LDH), before surgical procedure.

After staging, NSTGCT were more frequently found to have disseminated disease, as expressed by 57.4% of the patients opposed to 25% in the seminomatous group.

As expressed in Table 2, patients with STGCT were diagnosed with lower stages. A more heterogeneous distribution was found among nonseminomatous group. In this last group mixed tumors and embryonal carcinoma tumors were more prevalent.

**Table 2:** Histological Characteristics and Different Stages of Both TGCT Groups

Type	No. of cases	Stage								
		IA	IB	IS	IIA	IIB	IIC	IIIA	IIIB	IIIC
Seminomas	32	11	3	10	2	3	0	0	2	1
Nonseminomas	47	3	2	13	4	6	1	0	10	8
Embryonal Carcinoma	14	2	1	2	2	1	0	0	3	3
Mature Teratoma	1	0	0	1	0	0	0	0	0	0
Imature Teratoma	1	0	0	0	0	0	0	0	1	0
Choriocarcinoma	2	0	0	0	0	0	0	0	1	1
Mixed tumors	28	1	1	10	1	5	1	0	5	4
Teratocarcinoma	1	0	0	0	1	0	0	0	0	0

Risk classification and prognosis are listed in Table 3, conferring better prognosis among the seminomatous group.

**Table 3:** Risk Classification

	Risk Classification				
	Good		Intermediate		Poor
	STGCT	NSTGCT	STGCT	NSTCGT	NSTGCT
No residual disease	29	23	1	4	1
Residual disease	0	5	1	5	5
Progression	1	1	0	2	1

All the patients in both TGCT groups underwent radical orchiectomy. Complementary therapy is as described in Table 4. Radiotherapy was done in lower stages of the disease and chemotherapy was done in all the patients in stages II and III but one in stage IIA in the group of nonseminomatous (this patient was temporarily lost after surgery by CHP).

**Table 4:** Global Treatment Modalities Applied in Different Stages of TGCT. CT – Chemotherapy, RT - Radiotherapy

Stage	Seminomatous			Nonseminomatous		
	CT	RT	Total of patients	CT	RT	Total of patients
IA	1	5	11	1	1	3
IB	3	-	3	2	-	2
IS	8	2	10	8	1	13
IIA	2	-	2	3	-	4
IIB	3	-	3	6	-	6
IIC	-	-	-	1	-	1
IIIB	2	-	2	10	-	10
IIIC	1	-	1	8	-	8

CT: Chemotherapy; RT: Radiotherapy

After the initial approach (surveillance or complementary therapy), a re-evaluation was done. In the seminomatous group, only one patient with a previous stage IA progressed and another one with IIIB stage had residual disease. In the nonseminomatous group, one patient with IIA stage progressed (cited above). The same happened to two patients of IIIB and one IIIC stages. In the nonseminomatous group, four patients with IIB, one patient IIC, five IIIB and another five patients with IIIC stages had residual disease.

Intermediate and poor risk patients with nonseminomatous TGCT had a tendency to have residual or progression of the disease, as demonstrated by 63.3% (7/11 patients) and 85.7% (6/7 patients) respectively (Table 3).

After re-evaluation, residual disease was found in 16 (20.25%) patients in both groups, all of them presenting retroperitoneal conglomerate. Ten of them underwent retroperitoneal lymph node dissection (RPLND). One received additional chemotherapy (CT) and RPLND, another two just CT, one radiotherapy (RT) and two of them were kept under a surveillance program without any other treatment. It is of notice that from the 16 patients with residual disease, 15 had NSTGCT.

Fifty-eight patients after re-evaluation were considered free of disease, 30 of them (93.75%) had STGCT and 28 (59.6%) had NSTGCT. Four of them had a recurrence after an average of 19.25 months (minimum of 10 and maximum 39 months). Two were seminomas (stages IS and IA) and the other ones nonseminomas (IS and IIIC). Retroperitoneum and liver were the organs where metastasis was found.

Adverse effects attributed to medical treatment were found in 30 patients. Hematologic toxicity was the most prevailing one at 44.3% (27 out of 61 patients that received chemotherapy), lung at 4.08%, dermatologic at 3.3% and raynaud phenomena in 3.3%.

At the end of the study 89.9% of the patients were still alive. Eight patients died, four of them with NSTGCT. Death was attributed to cancer, two not related to TGCT and the other two related to a complication of the treatment.

In almost 1/3 of all patients (27.8%) a testicular prosthesis was implemented and semen cryopreservation was done in 30.4%.

**Figure 2:**
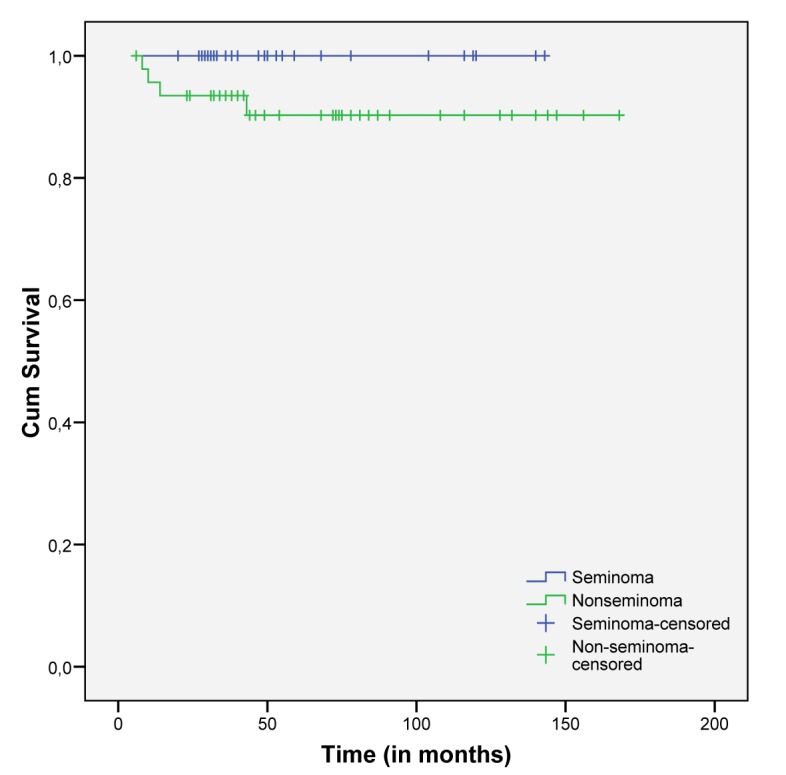
Cumulative survival in both subgroups of TGCT.

Overall survival was lower in NSTGCT but with no statistical significance (p=0.102), although all patients with STGCT were still alive (Fig. 2).

**Figure 3:**
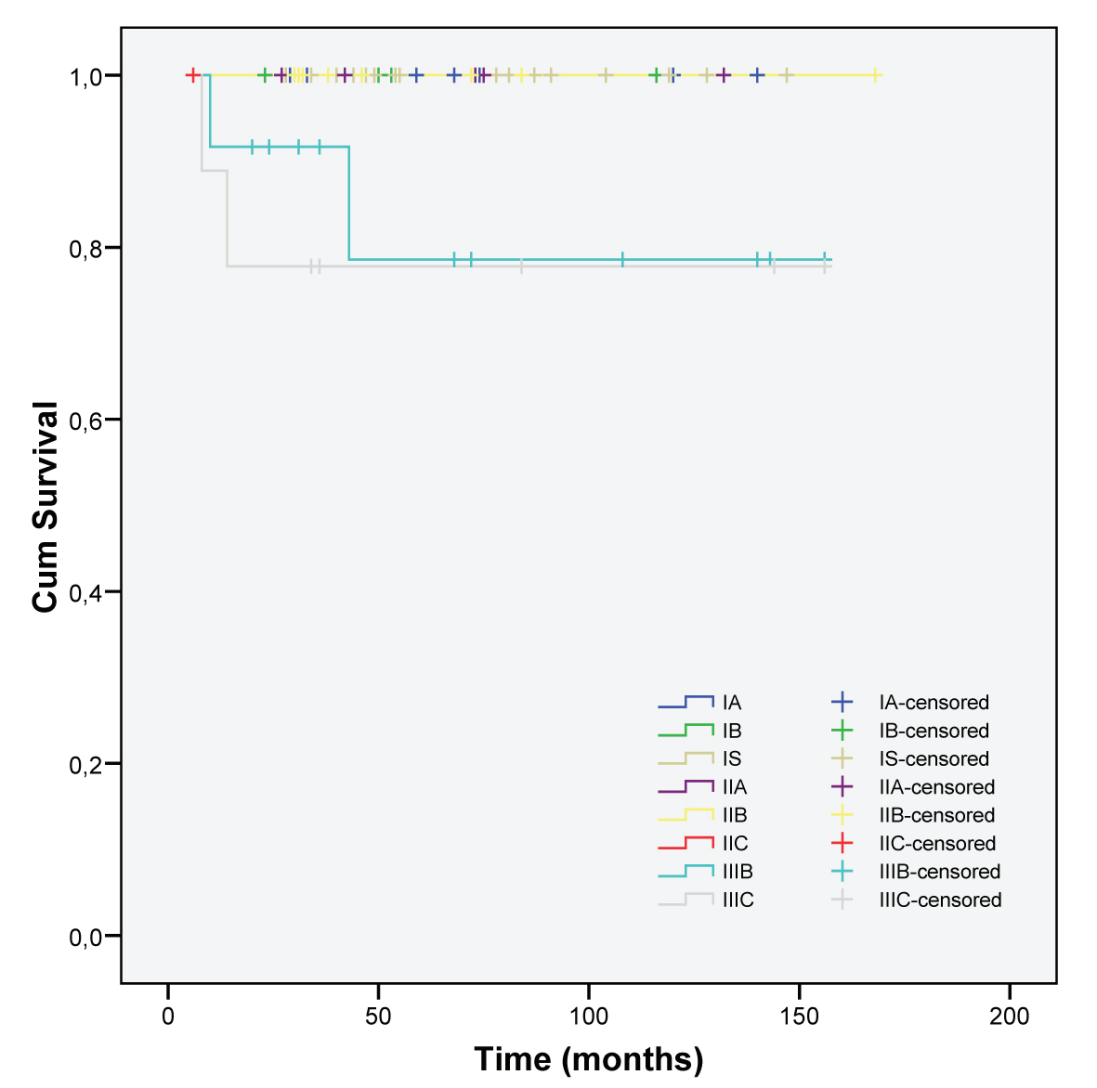
Cumulative survival in all initial stages.

**Figure 4:**
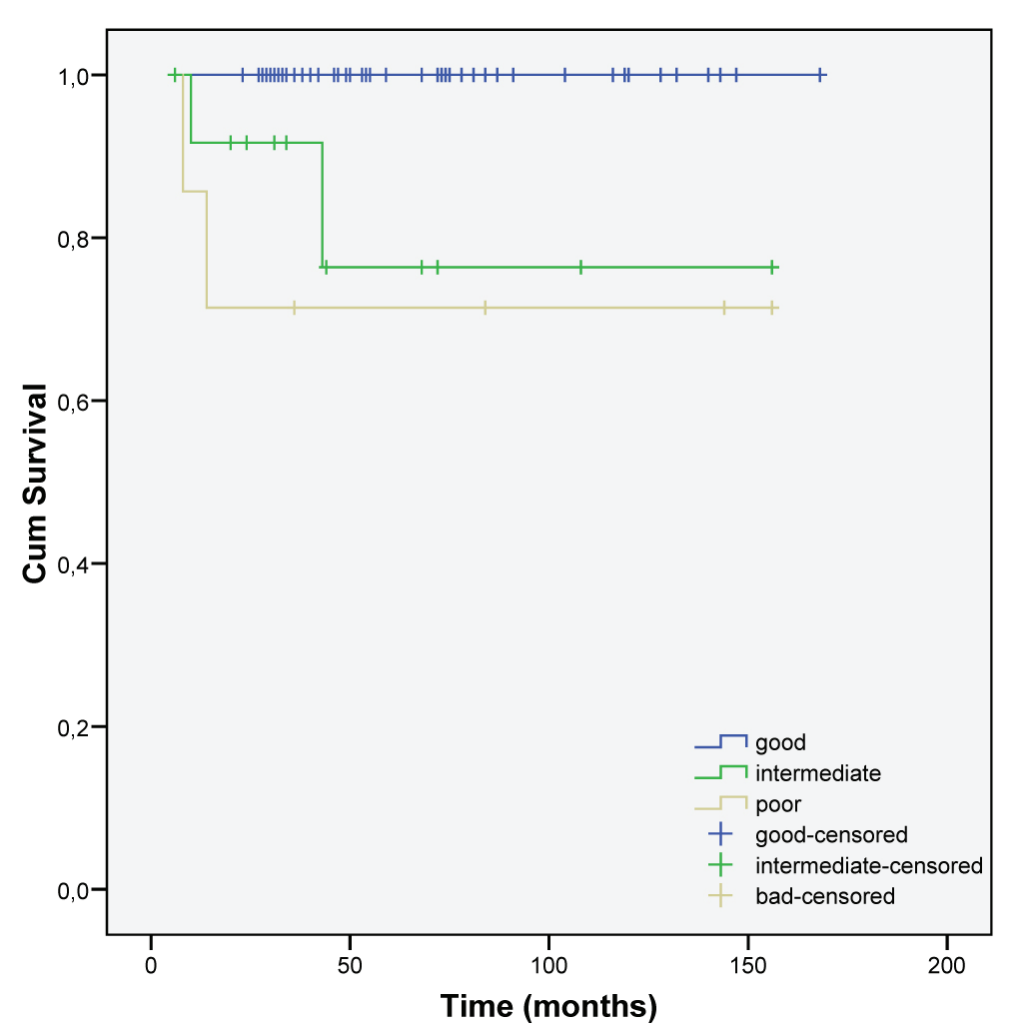
Cumulative survival according to risk score stratification.

More advanced stages were related to a low survival (intermediate and poor risk) (Fig. 3 and 4).

## Discussion

To our knowledge, this is the first report ever made in Portugal about clinical features, treatment and outcome in testicular tumors.

TGCT has been more frequently diagnosed on males in their twenties and thirties, mirroring the Western Europe incidence, with no significant differences between the two histological types [2].

Testicular microlithiasis may be a risk factor for the development of TGCT, but the association between this finding and testicular cancer is highly variable depending on the studies published [7]. A prospective study found only one (1.6%) out of 63 patients with microlithiasis as having developed a NSTGCT, and it concluded that self-examination should be done instead of an intensive screening imagery surveillance program [8]. In our population with TGCT we found 20.3% of patients with testicular microlithiasis. Its meaning as a risk factor for the development of TGCT may be questionable. As we don’t have the prevalence of testicular microlithiasis in the Portuguese healthy male population, this finding on 1/5 of the patients with TGCT should be interpreted as frequent association with TGCT.

Six patients (7. 6%) were found to be HCV and two HIV positive. It is known that HIV increases the incidence of TGCT, but there are no studies concerning the implication of HCV in testicular tumorogenesis [9]. Metachronous tumors were found in two patients, with the second tumor developing more than five years after the first one, as described elsewhere. This leads to a recommendation for long-term monitoring [10].

The most frequent clinical features at diagnosis were the localized ones such as pain and swelling testis, with similar prevalences among both groups. It was in the nonseminomatous group that symptoms of disseminated disease were most frequent. They presented a more severe features and a worse risk stratification that correlates with poor survival.

In spite of its after-the-fact approach, it was possible to access to all imagery and biochemical exams (testicular echography and CT scans) done at the time of diagnosis and before the surgical procedures, and it does demonstrate good compliance with the institution protocol.

The finding of a more severe disease by the time of presentation revealed by the patients symptoms in the nonseminomatous subgroup was confirmed after the initial staging. This was demonstrated by more than doubling of the disseminated disease in this group and a worse outcome.

STGCT presented with lower stages of disease and nonseminomatous tumors have a wider distribution. Higher stages are found more frequently in this subgroup.

Therapeutic options for treatment of TGCT patients have not changed much over recent years. This retrospective review of 12 years of experience also demonstrated this reality.

In the early stages (clinical stage I) of STGCT radiotherapy, chemotherapy or surveillance after initial diagnostic and therapeutic orchiectomy are all options to be considered [11]. Of the 24 patients in stage I, seven of them underwent adjuvant radiotherapy (20 to 30 Gy), ten received single carboplatin cycle, one BEPx3, one two cycles of carboplatin and five patients got only regular surveillance. None of the patients that were submitted to RT or carboplatin single cycle have relapsed or died, with mean surveillance follow-up of 68.0 ± 34.70 and 40.0 ± 14.70 months respectively. Both patients that were submitted to more aggressive chemotherapeutic schemes had vascular or spermatic cord invasion. One of them (stage IS) relapsed after 59 months, with visceral metastasis. A salvage treatment was done with BEP, but the patient died one month after metastatic disease was recognized. This was related to chemotherapy complications.

Nowadays, RT is not an option for early NSTGCT, but adjuvant chemotherapy or even tight surveillance may be [12]. From the 18 patients in this subgroup, six underwent BEPx3, four BEPx2, one received single carboplatin cycle and five were only subject to surgical procedure. Two patients received radiotherapy after orchiectomy in the late 1990’s, and both were still alive. In those days this RT treatment type was acceptable. Whatever type of treatment provided, the clinical response was similar. Cure rates were at 100%.

In 34 patients with retroperitoneal lymph node metastasis at presentation, confirming more advanced clinical stages, and after initial treatment (orchiectomy, chemotherapy, and orchiectomy with concomitant RPLND in only one patient), re-evaluation showed 14 were free of disease and sixteen with residual disease. After the initial chemotherapy 11 patients with residual disease underwent RPLND, as recommended. Two (18.2%) of them died but none of the deaths was related to disease progression. So, all the patients that underwent RPLND had their malignancy controlled, without relapse, conferring better prognosis than what is described elsewhere (risk of relapse in similar patients from 12 to 45%) [13].

Adverse side effects attributed to medical treatment are probably underestimated. The most frequently found were hematologic toxicity in 43.3%, and pulmonary toxicity in 4.08% of patients, attributed to bleomycin (49 patients received chemotherapy schemes with bleomycin sometime over the course of the disease). This is in line with what is described in literature [14].

Disease free survival rate is globally estimated to be 88.6%, with a median time of surveillance of 46 ± 34.7 months. Death attributed to testicular cancer (5.06%) happened only in the nonseminomatous group, and in patients with advanced initial stages (IIIB and IIIC). Prognosis of our patients was clearly influenced by the initial stage, as expected [15].

The promptness of the starting of treatment (as measured by a median of 8 days (IQR = 4 - 21.5) from the referral of patients until the start of treatment ) may have impacted the good outcome. Unfortunately, seeking a doctor’s help at the beginning of the symptoms is not so prompt, with a median of 21 days (IQR = 3 - 60), and that is probably an underestimation.

We don’t know if HCV infection has any role in tumorigenesis of TGCT. It could be just a simple coincidence, but it may be a future direction for other studies.

NSTGCT presented with worse prognosis, more advanced disease at presentation and survival rates that are lower than the STGCT subgroup. This later one had an excellent outcome independent of the stage or risk group stratification.

In conclusion, public health campaigns should be carried out to alert and teach how to make a testicular self exam to reduce the time from the beginning of symptoms and the time of seeking expert help. This may well improve the outcomes and cure rates.
